# Vitamin D status and muscle strength in a pan-European cohort of children and adolescents with normal weight and overweight/obesity

**DOI:** 10.1007/s00431-025-06024-9

**Published:** 2025-02-11

**Authors:** Hajo Zeeb, Tilman Brand, Lauren Lissner, Fabio Lauria, Dénes Molnár, Toomas Veidebaum, Matthias Nauck, Michael Tornaritis, Stefaan De Henauw, Luis A. Moreno, Wolfgang Ahrens, Hermann Pohlabeln, Maike Wolters, On behalf of IDEFICS and I.Family consortia

**Affiliations:** 1 Leibniz Institute for Prevention Research and Epidemiology – BIPS, Achterstrasse 30, 28359 Bremen, Germany; 2 Human and Health Sciences, University of Bremen, Bremen, Germany; 3 School of Public Health and Community Medicine, Institute of Medicine, Sahlgrenska Academy, University of Gothenburg, Gothenburg, Sweden; 4 Institute of Food Sciences, National Research Council, Avellino, Italy; 5 Department of Paediatrics, Medical School, University of Pécs, Pécs, Hungary; 6 National Institute for Health Development, Tallinn, Estonia; 7 Institute of Clinical Chemistry and Laboratory Medicine, University Medicine Greifswald, Greifswald, Germany; 8 DZHK (German Centre for Cardiovascular Research), University Medicine Greifswald, Partner Site Greifswald, Greifswald, Germany; 9 Research and Education Institute of Child Health, Strovolos, Cyprus; 10 Department of Public Health and Primary Care, Ghent University, Ghent, Belgium; 11 GENUD (Growth, Exercise, Nutrition and Development) Research Group, Instituto de Investigación Sanitaria de Aragón (IIS Aragón, University of Zaragoza, Instituto Agroalimentario de Aragón (IA2), Saragossa, Spain; 12 Centro de Investigación Biomédica en Red de Fisiopatología de La Obesidad y Nutrición (CIBERObn), Instituto de Salud Carlos III, Madrid, Spain

**Keywords:** Children cohort, Vitamin D, Handgrip strength, Overweight, Obesity

## Abstract

This aimed to investigate associations between the vitamin D status and handgrip strength in children and adolescents considering weight status. Participants aged 7 to < 16 years from the European IDEFICS/I.Family cohort were included. Serum 25-hydroxyvitamin D [25(OH)D] and handgrip strength were measured in 2013/2014. In multivariable logistic regression models, we investigated the association of vitamin D status with handgrip strength adjusted for demographic variables, BMI z-score, sports club membership, screen time and UV intensity. Vitamin D-sufficient children had higher odds for a high handgrip strength (OR = 1.92, 95%-CI: 1.12, 3.30), but this association was limited to children with thinness/normal weight (OR = 2.60, 95%-CI: 1.41–4.81). Children with overweight (OR = 2.64, 95%-CI: 2.00, 3.49) and obesity (OR = 4.53, 95%-CI: 2.93, 7.02) were more likely to have a high handgrip strength than thin/normal weight children. *Conclusions*: The positive association of vitamin D with muscle strength in our study is limited to children with low and normal BMI. In children with overweight or obesity, the higher muscle strength seems to superimpose the association with vitamin D. Overall, our results indicate the importance of sufficient levels of vitamin D and offers physical activity perspectives for children with overweight and obesity.

*Study registration*: ISRCTN: 10.1186/ISRCTN62310987.

**Table:** 

***What is known***: • *Studies have shown a positive association between vitamin D status and handgrip strength in older adults while studies in children and adolescents are scarce.* • *Conflicting results were found on whether overweight and obesity are associated with reduced or increased handgrip strength compared to normal weight.*	
***What is new:*** • *The results of this large pan-European study in children and adolescents indicate that a sufficient vitamin D status is associated with better handgrip strength, particularly in participants with thinness/normal weight.* • *Children with overweight and obesity have a higher handgrip strength as compared to children with thinness/normal weight which seems to superimpose the positive association between vitamin D status and handgrip strength in this group.*

**Supplementary Information:**

The online version contains supplementary material available at 10.1007/s00431-025-06024-9.

## Introduction

Evidence both from clinical and epidemiological studies underlines that vitamin D is important for normal development and growth; however, vitamin D deficiency is considered a global problem [1]. Vitamin D is linked to muscle strength across the life course [2], but the existence and nature of this link in childhood and adolescence remains conflicting [3]. There are numerous pathways through which vitamin D may be associated with muscle strength, notably via the presence of vitamin D receptors in skeletal muscle, via calcium and phosphate metabolism and/or cellular signalling [2,4].

Studies in adults, including older adults, have shown beneficial effects of vitamin D supplementation on muscle strength and handgrip strength [5,6] and a positive effect was also reported in younger adults [7–9]. However, not all studies confirmed favourable effects in older adults [10,11] or adults of all age groups [12]. There is also some evidence that a high/sufficient vitamin D status plays a beneficial role in the muscle and handgrip strength among children and adolescents. While some observational studies found a positive association between the vitamin D status and handgrip strength [13,14], others did not [15,16], and intervention studies mostly reported no effect of vitamin D supplementation on handgrip strength [17–20]. However, large multi-national studies conducted in this population are missing and the current evidence based on national studies is contradictory.

Thus, the pan-European IDEFICS/I.Family study provides an opportunity to further investigate the question of vitamin D status and muscle strength in children and adolescents. We aimed to assess the association between 25(OH)D concentrations of European children and their muscle strength measured by handgrip strength considering the role of the weight status.

## Materials and methods

This study included children and adolescents of the IDEFICS/I.Family cohort from eight European countries (Belgium, Cyprus, Estonia, Germany, Hungary, Italy, Spain and Sweden). In the baseline survey (Wave 1 = 2007/2008), 16,229 children aged 2–9 years participated and follow-up examinations were conducted 2 years (Wave 2 = 2010/2011) and 6 years later (wave 3 = 2013/2014). Data from a subsample of wave 3 was analysed in the present study. Detailed information on the study design can be found elsewhere [21].

Serum 25-hydroxyvitamin D [25(OH)D] concentrations were measured using a chemiluminescence assay as previously reported [22]. Vitamin D status was categorized as deficient (< 50 nmol/l), insufficient (50– < 75 nmol/l) or sufficient (≥ 75 nmol/l) [23]. The first two categories were also combined as “low vitamin D status.”

Handgrip strength test was assessed with a dynameter (TKK 5101, Takei, Tokyo, Japan), using the average of the right and left handgrip strength score, with results recorded in kilogram force (kgf). A higher score indicates a better performance.

Body weight and height were measured as reported elsewhere [22]. Percentage of fat free mass (%FFM) was assessed using hand-to-foot bioelectrical impedance analysis (TANITA BC 418 MA, Tanita Europe GmbH, Sindelfingen, Germany). Age- and sex-specific z-scores of handgrip strength were calculated based on previously published reference curves [24] and BMI reference standards [25] were applied to calculate age- and sex-specific BMI z-scores.

UV-B radiation was estimated based on UV dose data derived from atmospheric satellite data with further adjustment of cloud cover. Place-, year- and month-specific cloud-modified UV doses of the second last month before blood draw at (or near) the place of residence of the participant (UVDVC) were used [22]. Parental education as per the International Standard Classification of Education (ISCED), sports club membership (y/n) and weekly screen time (hours) were included as adjustment variables.

### Statistical analyses

The association between vitamin D and handgrip strength was analysed for the entire study group and separately for children with thinness/normal weight and children with overweight/obesity. Similarly, the association between weight status and handgrip strength was analysed separately for children with low (deficient or insufficient) and sufficient vitamin D status. Handgrip strength was categorized in low, medium and high based on the < 20th, 20–80th and > 80th age- and sex-specific reference percentiles, respectively. We used a multivariable logistic regression [outcome: handgrip strength > 80th percentile, interesting exposure: sufficient vitamin D = 1, low (deficient and insufficient) vitamin D = 0]. Model 1 was adjusted for only sex, age and country. Model 2 was additionally adjusted for membership in a sports club, screen time/week, BMI z-score, ISCED and UVDVC. Analyses were conducted using SAS 9.4 (Cary, NC). As multicollinearity can lead to problems in interpreting the coefficients of independent variables in a regression model, we analysed this for the association of serum 25(OH)D and UVDVC by calculating the variance inflation factor (VIF) by means of the corresponding option in PROC REG (SAS). We performed a supplementary analysis to check whether our data showed seasonal effects similar to those recently shown in another study [26] which indicated that the vitamin D status modulated physical performance in interaction with seasonality. To adequately account for this interaction in our logistic regression model, we stratified vitamin D status by season in this analysis.

## Results

A total of 1828 children and adolescents were included in the analysis (Table 1). One quarter of the children had strong handgrip strength above the 80th percentile while 16% showed a low strength. Only 4.2% of the children had a sufficient vitamin D status. Among children with obesity, deficient status was more prevalent (*n* = 83 out of 107, 77.6%) compared to children with thinness/normal weight (*n* = 840 out of 1398, 60.1%) or overweight (*n* = 223 out of 323, 69.0%). Children with a sufficient vitamin D status seemed to have a slightly higher (57.9 ± 29.1) mean handgrip percentile compared to children with a deficient (54.7 ± 29.5) or insufficient (52.6 ± 28.3) status (Table 1).

**Table 1:** Characteristics of the study population (*N* = 1828)

Parameters		*N*	% or mean (SD)
Sex, female		1828	48.5%
Age, years		1828	12.0 (1.8)
Age category	7– < 10 years	319	17.5%
	10– < 14 years	1249	68.3%
	14– < 16 years	260	14.2%
Study region	Belgium	38	2.1%
	Cyprus	29	1.6%
	Estonia	314	17.2%
	Germany	323	17.7%
	Hungary	406	22.2%
	Italy	250	13.7%
	Spain	250	13.7%
	Sweden	218	11.9%
Handgrip strength, kgf		1828	20.5 (6.8)
Handgrip strength, SI		1828	200.8 (66.6)
Handgrip strength z-score		1828	0.2 (1.0)
Handgrip strength category^a^	< 20th percentile	291	15.9%
	20–80th percentile	1078	59.0%
	> 80th percentile	459	25.1%
25(OH)D, nmol/l		1828	18.4 (7.2)
Vitamin D status^b^	Deficient	1146	62.7%
	Insufficient	605	33.1%
	Sufficient	77	4.2%
Mean 25(OH)D concentration (nmol/l) and handgrip strength percentile (hand grip *P*) by vitamin D status	*Deficient vitamin D*: 25(OH)D concentration Handgrip strength *P*	1146 1146	14.3 (3.8) 54.7 (29.5)
	*Insufficient vitamin D*: 25(OH)D concentration Handgrip strength P	605 605	23.6 (2.7) 52.6 (28.3)
	*Sufficient vitamin D*: 25(OH)D concentration Handgrip strength *P*	77 77	38.0 (9.9) 57.9 (29.1)
BMI (kg/m^2^)		1828	19.5 (3.8)
BMI z-score^c^		1828	0.5 (1.1)
Weight status^c^	Thin/normal weight	1398	76.5%
	Overweight	323	17.7%
	Obese	107	5.9%
Weight status^c^ by vitamin D status	Low vitamin D status^b,d^ and thin/normal weight	1,337	73.1%
	Low vitamin D status^b,d^ and overweight/obese	414	22.7%
	Suff. vitamin D status^b^ and thin/normal weight	61	3.3%
	Suff. vitamin D status^b^ and overweight/obese	16	0.9%
Parental education based on ISCED^e^	Low	77	4.2%
	Medium	806	44.1%
	High	925	50.6%
	Missing	20	1.1%
Sports club member	Yes	1236	67.6%
	No	535	29.3%
	Missing	57	3.1%
Screen time, hours/week		1720	16.7 (10.5)
UV exposure (UVDVC), kJ/m^2^		1828	2.1 (2.1)
% fat free mass		1818	77.3 (6.4)

^a^Handgrip strength percentiles were calculated based on previously published reference percentiles (De Miguel-Etayo et al. [24])

^b^Vitamin D status based on serum 25(OH)D: deficient (< 50 nmol/l), insufficient (50– < 75 nmol/l), sufficient (≥ 75 nmol/l)

^c^Weight category for BMI was calculated using Cole and Lobstein [25]

^d^Low vitamin D status based on serum 25(OH)D defined as deficient (< 50 nmol/l) or insufficient (50– < 75 nmol/l)

^e^ISCED, International Standard Classification of Education; suff., sufficient; UVDVC, place-, year- and month-specific cloud-modified vitamin-D UV dose of the second last month before blood draw at (or near) the place of residence of the participant

In the fully adjusted regression model, children with a sufficient vitamin D status were about twice as likely to achieve a handgrip strength above the 80th percentile (OR = 1.92, 95%-CI: 1.12, 3.30) (Table 2). However, when this analysis was stratified according to weight status, this association was observed only in children with thinness/normal weight (OR = 2.60, 95%-CI: 1.41, 4.81). In children with overweight and obesity, however, there was no difference in handgrip strength according to vitamin D status (OR = 0.66, 95%-CI: 0.21, 2.09). Additional adjustment for fat free mass slightly increased the odds ratios, in the complete sample (OR = 2.04, 95%-CI: 1.16, 3.58) as well as in children with thinness/normal weight (OR = 2.75, 95%-CI: 1.46, 5.18) and in children with overweight/obesity (OR = 0.73, 95%-CI: 0.22, 2.49). There was no indication of multicollinearity.

**Table 2:** Odds ratio estimates and 95% confidence intervals for associations of vitamin D status with handgrip strength percentiles by weight status and by vitamin D status

	Handgrip strength		Odds ratio estimate (OR) (95% confidence interval)	
	≤ 80th percentile	> 80th percentile	Crude^c^	Adjusted^d^
Complete sample	*N* (%)	*N* (%)	OR 1	OR 2
Low vitamin D status^a^	1319 (75.3%)	432 (24.7%)	1	1
Sufficient vitamin D status^a^	50 (64.9%)	27 (35.1%)	1.63 (0.98, 2.69)	1.92 (1.12, 3.30)
Thin and normal weight children^b^				
Low vitamin D status^a^	1071 (80.1%)	266 (19.9%)	1	1
Sufficient vitamin D status^a^	40 (65.6%)	21 (34.4%)	2.13 (1.20, 3.77)	2.60 (1.41, 4.81)
Overweight and obese children^b^				
Low vitamin D status^a^	248 (59.9%)	166 (40.1%)	1	1
Sufficient vitamin D status^a^	10 (62.5%)	6 (37.5%)	0.64 (0.21, 1.94)	0.66 (0.21, 2.09)
Complete sample	*N* (%)	*N* (%)	OR 1	OR 2^e^
Underweight/normal weight	1111 (79.5%)	287 (20.5%)	1	1
Overweight	204 (63.2%)	119 (36.8%)	2.61 (1.98, 3.44)	2.64 (2.00, 3.49)
Obese	54 (50.5%)	53 (49.5%)	4.49 (2.93, 6.89)	4.53 (2.93, 7.02)
Low vitamin D status^a^				
Underweight/normal weight	1071 (80.1%)	266 (19.9%)	1	1
Overweight	196 (63.2%)	114 (36.8%)	2.71 (2.04, 3.60)	2.77 (2.08, 3.69)
Obese	52 (50.0%)	52 (50.0%)	4.84 (3.13, 7.47)	4.98 (3.18, 7.79)
Children with sufficient vitamin D status^a^				
Underweight/normal weight	40 (65.6%)	21 (34.4%)	1	1
Overweight	8 (61.5%)	5 (38.5%)	1.35 (0.35, 5.12)	1.33 (0.30, 5.82)
Obese	2 (66.7%)	1 (33.3%)	0.85 (0.07, 10.9)	0.61 (0.04, 8.97)

^a^Vitamin D status based on serum 25(OH)D defined as low: deficient (< 50 nmol/l) or insufficient (50– < 75 nmol/l); sufficient (≥ 75 nmol/l)

^b^Weight category for BMI was calculated using Cole and Lobstein^25^

^c^Crude model is adjusted for age, sex and country

^d^Adjusted model is additionally adjusted for membership in sports club, screen time/week, BMI z-score, UV exposure and parental education status

^e^Odds ratios for weight status in this model are not additionally adjusted for BMI z-score

Children with overweight were more than twice as likely (OR = 2.64, 95%-CI: 2.00, 3.49) and children with obesity were more than four times as likely (OR = 4.53, 95%-CI: 2.93, 7.02) to score above the 80th percentile of handgrip strength as compared to children with thinness/normal weight. Similar results were observed if only children with a low vitamin D status were considered (Table 2).

In the supplementary analysis to assess a potential interaction of the vitamin D status with seasonality, the descriptive data already indicated a trend of an effect of seasonality (Table S1). In children with sufficient vitamin D status, handgrip strength is on average greater in spring (61.9) and autumn (59.6) compared to summer (49.4) and winter (47.0). In the logistic regression analysis, there was a very low number of children measured in winter with a sufficient vitamin D status (*N* = 4). Therefore, we combined all children measured in winter into a single group. Our results indicated that the effect of sufficient vitamin D on handgrip strength was more pronounced in children measured in spring (OR = 2.71; 95%-CI: 0.71, 10.4) or in autumn (OR = 1.52; 95%-CI: 0.73, 3.18) than in children measured in summer (OR = 1.00; 95%-CI: 0.18, 5.67), each compared to children measured in winter (Table S2).

## Discussion

Our results show that children with a sufficient vitamin D status were twice as likely to reach a high handgrip strength compared to children with a low vitamin D status. This association was not observed in children with overweight and obesity. However, these children anyway had a higher chance to have high handgrip strength as compared to children with thinness/normal weight, irrespective of vitamin D status. Thus, the lack of association of vitamin D status with handgrip strength in this subgroup could indicate that weight status has a stronger effect on handgrip strength than vitamin D, especially when considering that obesity has been shown to be associated with low vitamin D status [4,23]. Similar to our results, a study in Ethiopian school children found a positive association of serum 25(OH)D and handgrip strength independent of adiposity, age, sex, weight and height and also reported a positive association of weight and handgrip strength [14]. A study in New Zealand reported a positive association of serum 25(OH)D with handgrip strength independent of recreational physical activity and BMI in young women [8].

In contrast, most intervention studies did not support a positive effect of vitamin D supplementation on handgrip strength: An intervention study in 9–13-year-old children in the USA reported no effect of increased serum vitamin D metabolites on handgrip and muscle strength after a 12-week supplementation [20]. Similarly, a 24-week vitamin D supplementation trial in Danish children with a sufficiently high vitamin D status aged 6–8 years [19] and a 20-week intervention in 4–8-year-old Danish children with an insufficient status did not observe an effect on handgrip strength [18]. A 1-year vitamin D supplementation in Lebanese school girls aged 10–17 years with an insufficient vitamin D status also did not lead to a grip strength increase [17] although it has been suggested that a positive effect of vitamin D supplementation on muscle strength can only be achieved in participants with a deficient vitamin D status at baseline [20]. A UK vitamin D supplementation study in postmenarchal girls aged 12–14 years with a deficient baseline vitamin D status reported a non-significant increase of handgrip strength by 7.24% in the vitamin D group compared with the placebo group [27]. Globally, these studies may be seen to suggest that there is an association which may be mediated, for instance, by sedentary lifestyle, overall health, nutritional balance or body composition, but not a direct causal relationship between vitamin D status and muscle strength. On the other hand, it is conceivable that intervention studies do not show an effect because the intervention duration is often short and the dosage too low to achieve a sufficient vitamin D status [28,29].

We found overweight and obesity to be associated with higher handgrip strength. A strong influence of the weight status was also reported by others [30], and taken together, weight status seems to play an important role for handgrip strength. In contrast to previous studies, our data was obtained from a pan-European cohort including children and adolescents from Northern, Southern, Eastern and Western countries of Europe who had generally a low physical activity level [31]. As handgrip strength mainly represents forearm strength, the better performance of children with excess weight may help to motivate them to engage in sports where a strong forearm is particularly important such as martial arts, e.g. judo, climbing or tennis.

Our data indicated an interaction of the vitamin D status with seasonality resulting in higher handgrip strength in children measured in spring and autumn compared to winter. Due to small cell frequencies, these results need very careful interpretation. However, they are in line with effects previously described by Milani et al. [26] who found that compared to winter, spring or autumn provided a different effect of vitamin D on fitness performance. In this context, it is worth noting that an additional adjustment for season changed the odds ratios in our manuscript only minimally, so the results shown in Table 2 of the paper remain valid.

### Strengths and limitations

To our knowledge, this is the first pan-European study in children and adolescents investigating the association of vitamin D status and handgrip strength including a large number of measurements. We used age- and sex-specific z-scores of handgrip strength based on reference percentiles and additionally adjusted for the most important covariates and confounders. All measurements were conducted according to standardized protocols in all study centres. A limitation of our study is the small number of participants with a sufficient vitamin D status, particularly in the stratified analysis by weight status. The behavioural covariates were self-reported, and potential misreporting may have influenced the associations. Sports club membership and screen time were used as proxy measures for physical behaviour. Our definition of the vitamin D status was based on the recommendations of the Society for Adolescent Health and Medicine [23] while other authors and societies have proposed lower targets, e.g. sufficiency > 50 nmol, insufficiency 25–50 nmol/l, deficiency < 25 nmol/l [32,33]. Applying these cut-offs might have modified our results and conclusions.

## Conclusion

A better vitamin D status seems to be weakly associated with higher handgrip strength in children with a normal weight status, but not in children with overweight or obesity. Overweight and obesity are associated with higher handgrip strength as compared to thinness/normal weight which may help to motivate these children to engage in activities where they benefit from this strength and gain additional overall fitness. Public health measures should promote a sufficient vitamin D status, physical activity and a healthy weight in young populations.

## Supplementary Information

Below is the link to the electronic supplementary material.

Supplementary file1 (DOCX 30.6 KB)

## Data Availability

No datasets were generated during the current study.

## Abbreviations

25(OH)D: 25-Hydroxyvitamin D
BMI: Body mass index
ISCED: International Standard Classification of Education
OR: Odds ratio
SD: Standard deviation
UVDVC,: Place-, year- and month-specific cloud-modified vitamin-D UV dose of the second last month before blood draw at (or near) the place of residence of the participant
