# Supplementary material for: Vitamin D status and muscle strength in a pan-European cohort of children and adolescents with normal weight and overweight/obesity
Source: Eur J Pediatr. 2025 Feb 11;184(2):190. doi: 10.1007/s00431-025-06024-9 (PMC11814003; doi:10.1007/s00431-025-06024-9)
Supplement: Supplementary file 1 — Supplementary file1 (DOCX 30.6 KB) [file 431_2025_6024_MOESM1_ESM.docx]

Supplementary information to the article:

**Vitamin D status and muscle strength in a pan-European cohort of children and adolescents with normal weight and overweight/obesity**

Hajo Zeeb^1,2^, Tilman Brand^1^, Lauren Lissner^3^, Fabio Lauria^4^, Dénes Molnár^5^, Toomas Veidebaum^6^, Matthias Nauck^7,8^, Michael Tornaritis^9^, Stefaan De Henauw^10^, Luis A Moreno^11^, Wolfgang Ahrens^1^, Hermann Pohlabeln^1^, Maike Wolters^1*^

^1^Leibniz Institute for Prevention Research and Epidemiology – BIPS, Bremen, Germany

^2^Human and Health Sciences, University of Bremen, Bremen, Germany

^3^School of Public Health and Community Medicine, Institute of Medicine, Sahlgrenska Academy, University of Gothenburg, Gothenburg, Sweden

^4^Institute of Food Sciences, National Research Council, Avellino, Italy

^5^Department of Paediatrics, Medical School, University of Pécs, Pécs, Hungary

^6^National Institute for Health Development, Tallinn, Estonia

^7^Institute of Clinical Chemistry and Laboratory Medicine, University Medicine Greifswald, Greifswald, Germany

^8^DZHK (German Centre for Cardiovascular Research), Partner Site Greifswald, University Medicine Greifswald, Greifswald, Germany

^9^Research and Education Institute of Child Health, Strovolos, Cyprus

^10^Department of Public Health and Primary Care, Ghent University, Ghent, Belgium

^11^GENUD (Growth, Exercise, Nutrition and Development) Research Group, University of Zaragoza, Instituto Agroalimentario de Aragón (IA2), Instituto de Investigación Sanitaria de Aragón (IIS Aragón) Zaragoza, Spain and Centro de Investigación Biomédica en Red de Fisiopatología de la Obesidad y Nutrición (CIBERObn), Instituto de Salud Carlos III, Madrid, Spain

*on behalf of IDEFICS and I.Family consortia

**Corresponding Author:**

Hajo Zeeb, MD, PhD, ORCID ID: 0000-0001-7509-242X

Leibniz Institute for Prevention Research and Epidemiology - BIPS,

Tel: +49 421 218 56 902 Email: zeeb@leibniz-bips.de

**Supplementary analysis**

At the editors’ suggestion, we performed an additional analysis, to check whether our data revealed seasonal effects similar to those recently shown in a study published by Milani et al. (2021)^1^, according to which “... vitamin D levels modulated physical performance in interaction with seasonality...”.

**Table S1** shows the descriptive statistics for the variable handgrip percentile stratified by season and vitamin D status.

**Table S1:** Handgrip strength percentile (mean, standard deviation (SD) and median) stratified by season and sufficient or low vitamin D status

|  |  |  | **Handgrip strength percentile** | | |
| --- | --- | --- | --- | --- | --- |
| **Season** | **Vitamin D status** | **N** | **Mean** | **SD** | **Median** |
| Spring | Low | 573 | 51.3 | 29.7 | 51.9 |
|  | Sufficient | 10 | 61.9 | 23.9 | 59.7 |
| Summer | Low | 107 | 59.0 | 27.1 | 61.4 |
|  | Sufficient | 10 | 49.4 | 33.5 | 53.7 |
| Autumn | Low | 579 | 55.2 | 28.2 | 56.9 |
|  | Sufficient | 53 | 59.6 | 28.1 | 60.6 |
| Winter | Low | 492 | 54.5 | 29.8 | 56.8 |
|  | Sufficient | 4 | 47.0 | 46.4 | 47.7 |

^a^Vitamin D status based on serum 25(OH)D defined as low: deficient (<50 nmol/l) or insufficient (50-<75 nmol/l); sufficient (≥75 nmol/l)

As the number of children with sufficient vitamin status in our study is relatively small (n=77), this further stratification naturally results in even smaller numbers. Nevertheless, the trend described by Milani et al.^1^ is also supported by our data (**Table S1**). In children with sufficient vitamin D status, handgrip strength is on average greater in spring (61.9) and autumn (59.6) compared to summer (49.4) and winter (47.0).

To adequately account for this interaction in our logistic regression model, we stratified vitamin D status by season in a supplementary analysis. However, to obtain reasonably reliable parameter estimates it seems appropriate to have a minimum number of at least 10 exposed children per season, which is why the children measured in winter were combined into a single group.

**Table S2**: Odds ratio estimates and 95% confidence intervals for associations of vitamin D status with handgrip strength percentiles – stratified be the combined effects of season and vitamin D status

|  | | **Handgrip strength** | | **Odds ratio estimate (OR)**  **(95% Confidence interval)** | |
| --- | --- | --- | --- | --- | --- |
|  | | **<=80^th^ percentile** | **> 80^th^ percentile** | **Crude**^b^ | **Adjusted**^c^ |
| **Complete sample** | | **N (%)** | **N (%)** | **OR 1** | **OR 2** |
| Low vitamin D status^a^ | | 1,319 (75.3%) | 432 (24.7%) | 1 | 1 |
| Sufficient vitamin D status^a^ | | 50 (64.9%) | 27 (35.1%) | 1.63 (0.98, 2.69) | 1.92 (1.12, 3.30) |
| **Stratified by season and vitamin D status** | | |  | | |
| **Season** | **Vitamin D status^a^** |  | | | |
| Winter | Low or sufficient | 356 (71.8%) | 140 (28.2%) | 1 | 1 |
| Spring | Low | 458 (79.9%) | 115 (20.1%) | 0.66 (0.49, 0.88) | 0.77 (0.51, 0.99) |
|  | Sufficient | 6 (60.0%) | 4 (40.0%) | 1.95 (0.52, 7.32) | 2.71 (0.71, 10.4) |
| Summer | Low | 75 (70.1%) | 32 (29.9%) | 0.99 (0.61, 1.59) | 1.18 (0.59, 2.34) |
|  | Sufficient | 8 (80.0%) | 2 (20.0%) | 0.60 (0.12, 2.95) | 1.00 (0.18, 5.67) |
| Autumn | Low | 432 (74.6%) | 147 (25.4%) | 0.76 (0.57, 1.01) | 0.87 (0.58, 1.30) |
|  | Sufficient | 34 (64.2%) | 19 (35.8%) | 1.27 (0.68, 2.37) | 1.52 (0.73, 3.18) |
| Total | | 1,369 (74.9%) | 459 (25.1%) |  |  |

^a^Vitamin D status based on serum 25(OH)D defined as low: deficient (<50 nmol/l) or insufficient (50-<75 nmol/l); sufficient (≥75 nmol/l)

^b^Crude model is adjusted for age, sex and country;

^c^Adjusted model is additionally adjusted for membership in sport club, screen time/week, BMI z-score, UV exposure, and parental education status

The results in **Table S2** show that the effect of sufficient vitamin D status on handgrip strength was more pronounced in children measured in spring (OR=2.71; 95%-CI: 0.71-10.4) or in autumn (OR=1.52; 95%-CI: 0.73-3.18) than in children measured in summer (OR=1.00; 95%-CI: 0.18-5.67) – each compared to children measured winter.

**References**

1. Milani, G.P., Simonetti, G.D., Edefonti, V., Lava, S.A.G., Agostoni, C., Curti, M., Stettbacher, A., Bianchetti, M.G., and Muggli, F. (2021). Seasonal variability of the vitamin D effect on physical fitness in adolescents. Sci Rep 11, 182. 10.1038/s41598-020-80511-x.
